# Laparoscopic radical and partial cystectomy

**DOI:** 10.4103/0972-9941.19266

**Published:** 2005-10

**Authors:** Ben J. Challacombe, Kristen Rose, Prokar Dasgupta

**Affiliations:** Department of Urology, Guy's Hospital, London, United Kingdom

**Keywords:** laparoscopy, radical cystectomy, robotics, partial cystectomy

## Abstract

Radical cystectomy remains the standard treatment for muscle invasive organ confined bladder carcinoma. Laparoscopic radical cystoprostatectomy (LRC) is an advanced laparoscopic procedure that places significant demands on the patient and the surgeon alike. It is a prolonged procedure which includes several technical steps and requires highly developed laparoscopic skills including intra-corporeal suturing. Here we review the development of the technique, the indications, complications and outcomes. We also examine the potential benefits of robotic-assisted LRC and explore the indications and technique of laparoscopic partial cystectomy.

## INTRODUCTION

In the absence of randomized controlled trials, radical cystectomy is currently regarded as the gold standard treatment for muscle invasive, organ confined urothelial carcinoma of the bladder. It is also indicated for some patients with recurrent transitional cell CIS that have been unresponsive to intravesical chemotherapy. The goal of radical cystectomy is to achieve a surgical cure. In addition it may be performed as a palliative procedure to control severe disease related symptoms such as haematuria. Radical cystectomy has been shown to have better local disease control and 5-year survival than the other treatment options. Radical cystectomy has a significant complication rate approaching 25%, including mortality, even in the best hands. The median blood loss is often 1500–1800 ml and many patients subsequently require a blood transfusion.[1] The hospital stay and full recovery are consequently quite prolonged with 19–21 days recently reported as the standard UK average.[2] As experience with minimally invasive procedures continues to grow, urologists are able to expand laparoscopic indications. Laparoscopic radical cystectomy (LRC) is the result of this progress in laparoscopy and is a challenging procedure requiring advanced laparoscopic skills.[1,3]

### Evolution of LRC

Like most other laparoscopic procedures, LRC has evolved as a combination of procedures performed in animal models and patients over a decade. The initial case of laparoscopic cystectomy, reported in 1992, was for pyocystis in a 27 year old paraplegic woman whose bladder had been left behind after a supravesical urinary diversion a few months earlier.[4] Operative time was 130 min and postoperative hospital stay was only 5 days. By the following year, laparoscopic cystectomy with reconstruction using either ureterocutaneostomy or ureterosig-moidostomy had been successfully experimentally performed in Milan, Italy on ten pigs.[5] This was closely followed by the first LRC and extracorporeal ileal conduit for an invasive bladder tumour performed on a female patient by a Spanish group from Malaga.[6] Puppo et al then described anterior pelvic exenteration in 5 females through a combined transvaginal and laparoscopic approach in 1995. The specimen was removed trans-vaginally and operative time ranged from 6 to9 hours.[7] Despite this period of initial enthusiasm, no other centres commenced an LRC programme until 1999 when the team from Mansoura, Egypt published ten cases using a modified ureterosigmoidostomy diversion through a mini-laparotomy incision.[8] This series was not without its problems as they had a death from massive haemorrhage and also encountered external iliac artery clipping requiring vascular resection and anastomosis, a fistula which settled conservatively, a pelvic collection and a deep-vein thrombosis. However, as the leading institution for the treatment of schistosomiasis induced squamous cell carcinoma of the bladder, this centre has a higher proportion of younger patients with bladder cancer, making them ideally suited for longer complex procedures due to their relatively lower medical co-morbidities.

### Evolution of laparoscopic urinary diversion and neobladder formation

The Yale group experimented using an animal model to form a continent urinary diversion. They laparoscopically created a sigmoid-rectum (Mainz II) pouch and showed feasibility in 9 pigs but had problems with stone formation over the staple line in almost half of the animals.[9] In 2000, Kavoussi's group from Johns Hopkins published a five year follow-up on a laparoscopically created ileal conduit. This was performed using a 5-port transabdominal approach with a modified Bricker technique on a 28-year-old man with cerebral palsy and a neurogenic bladder requiring urinary diversion.[10] LRC and completely intracorporeal ileal conduit urinary diversion soon followed. Gill's group performed the procedures which took 10 and 11 hours, with an estimated blood loss of over 1 litre and a hospital stay of only 6 days.[11]

The next advancement was to mimic the open reconstructive procedures for neobladder formation after LRC. Turk's group from Berlin reported the initial continent urinary diversion using a sigma rectum (Mainz II) pouch in 2001.[12] At the same time, Gill's group from the Cleveland clinic were developing the technique of laparoscopic orthotopic neobladder; initially experimentally in pigs using a tubular Studer limb extension[13] and subsequently successfully in humans the following year.[14] This article also included a report of an Indiana pouch. Operative time was between 8.5 and 10.5 hours, with a blood loss ranging from 200 to 400 ml. Hospital stay was 5 to 12 days.

Gill's group have also described the technique of completely intracorporeal laparoscopic radical cystectomy in the female patient.[15] A variety of reconstructive techniques were successfully employed: an ileal conduit in 8 patients, Studer orthotopic neobladder in 2 and continent Indiana pouch in 1 with a good short-term functional and oncological outcome.

Interest has in LRC continues to rapidly increase and several centres have recently published larger series with good operative and oncological parameters (Table 1).[16–24] The range of reconstructive options is represented with one group reporting a hand-assisted technique as a learning aid prior to pure LRC.[19] Hemal has also described hand-assisted laparoscopic cystectomy[25] with a case successfully completed in 4.3 hrs and the patient discharged on the 7th day. The blood loss was 500 ml, and patient was transfused 2 units of blood, as the pre operative haemoglobin was 9gms/L. This technique has the advantage that after specimen retrieval, ileal conduit reconstruction can be performed extra-corporeally through the site of hand port incision. They conclude that the hand helps in retraction and performing blunt dissection coupled with tactile sensation. However this advantage needs to be balanced against the additional time required for set up and the added cost of the hand-port.

**Table 1:** Operative Outcomes

Authors	Reconstruction	N	Estimated blood loss, litres	Operative time, hr	Operative margins	Hospital stay, d	Complications
Puppo^7^1995	ICExtra-corporeal	5	3 transfused 2-6 units	6-9	NS	7-11	none
Denewer^8^1999	MUExtra-corporeal	10	2.2 units (2-3)	3.6 (3.2-4.1)	NS	10-13	1 × death (bleeding) 1 × external iliac artery clipped1 × urine leak, 1 × DVT1 × pelvic collection
Gill^11^2000	ICIntra-corporeal	2	1.1 (1.0-1.2)	10.8 (10-11.5)	All −ve	6	none
Gupta^23^2002	IC Intra-corporeal	5	0.36 (0.3-0.4)	7.5 (7-8)	All −ve;2 node+ve	7 (6-22)	1 × bowel obstruction1 × abdominal distension
Turk^12^2001	MUIntra- corporeal	5	0.25 (0.19-0.3)	7.4 (6.9-7.9)	All −ve	10	none
Gill^14^2002	OIN/IPIntra/Extra	2/1	0.3/0.3	9.5/7	All −ve	8.5 (5-12)/6	Bleeding duodenal ulcer
Hemal 242003	IC Extra-corporeal	10	0.53	6.5	1/10 positive	10.8	1 × open conversion 5 major/minor
Menon 292003	OIN/IC Extra	17	<0.15	5.1/4.3	All −ve	NS	1 × open exploration for bleeding.1 corporeal Robot assist × conversion for optic problem
Cathelineau1^16^ 2005	33 IC51 OIN Extra-corporeal	84	0.5 (0.15-2.0)	4.7 (3.6-5.5)	All −ve	12 (8-31)	2 × urinary fistulas3 × haematomas1 × pyelonephritis1 × PE 8 × urinary infections
DeGer^21^2004	OIN/IC/MU5/3/12 Intra corporeal	20	0.2 (0.19-0.8)	8.1 (6.1-13.8)	All −ve; 3	15 (11-30)	1 × persistent lymph node +ve leakage of the pouch1 × rectovaginal fistulaboth needed re-operation
Yang^19^2005	ICHA 11/Lap 7	18	286/179	6.5/6.0	NS	7	no major complications
Simonato^17^ 2005	OIN/US/CU6/2/2 Extra-corporeal	10	0.31 (0.2-0.40)	9.8 (8.5-10.5)	All −ve	8 (7-9) 5 8 (7-9)
Huang^20^2005	Orthotopic neobladder Extra-corporeal	33	0.46 mean	6.5 hours	All −ve		6/33 (18%)2 × pouch leakage2 × bowel obstruction1 × fistula1 × pelvic infection

Abbreviations: IC: Ileal Conduit, MU: modified ureterosigmoidostomy, OIN: Orthotopic Ileal neobladder, IP: Indiana Pouch, CU: Cutaneous urostomy, US: Ureterosigmoidostomy, HA: hand-assisted, PE: pulmonary embolism, DVT: Deep vein thrombosis, NS: not stated

### The technique of LRC

As for the majority of laparoscopic procedures, the operative steps mimic those of the equivalent open operation, however the technique is still in evolution and differences exist between operating surgeons. From our own experience and that of others, it is evident that a team approach is essential to the success of this complex procedure. As it is a prolonged procedure, it appears to be ideal for two experienced surgeons to be operating together so that different sections of the procedure can be shared leading to less surgical fatigue and improving outcomes.

Under a general anaesthetic, the patient is placed in a supine lithotomy position with the operative table in a 45° Trendelenberg tilt. Extra care must be taken to adequately protect potential pressure areas with appropriate padding and bilateral leg compression stockings and flowtron boots should be applied to prevent deep vein thrombosis and compartment syndrome. The anaesthetist needs to be warned about this position so as to reduce any risk of ventilatory difficulties.

Most surgeons use a 5/6 port trans-peritoneal approach and these are placed in a similar semi-circular manner similar to laparoscopic radical prostatectomy. Many centres perform an extended bilateral pelvic lymphadenectomy at this stage but we prefer to leave this till after the cystectomy. The peritoneum is incised transversely developing the pouch of Douglas and allowing dissection and division of the vas deferentia. Following incision of Denonvillier's fascia the plane posterior to the prostate and anterior to the rectum is developed. A rectal balloon or sigmoidoscope can sometimes help with identification of this fat plane and prevent rectal injury.

At this stage the ureters are identified, clipped and divided with the distal margins sent for frozen section histological analysis. The lateral pedicles are then developed by dissection medial to the obturator fossa. These pedicles are then controlled either with a series of locking clips, endovascular staplers or harmonic scalpel. Clips are the cheapest options but bleeding is somewhat more than the other two techniques. Staplers are the most expensive as multiple firings of cartridges are required. In our opinion, a cost-effective and efficient method is the use of a harmonic scalpel. Newer generations of this device work much faster than the older ones and are good value for money.

The peritoneum is divided anteriorly to drop the bladder down. Filling the bladder with 250 ml of formol-saline can help with this step. The entire urachus is detached and the retropubic space developed. After incising the endopelvic fascia and dividing the puboprostatic ligaments, the dorsal vein is controlled using one or several interrupted sutures. Occasionally an endovascular stapler or harmonic scalpel can be used to control the dorsal vein but in our experience, intracorporeal suturing provides the most secure haemostasis. At this stage cavernous nerve sparing can be performed in patients wishing to preserve potency, as in radical prostatectomy. The urethra is then divided carefully to avoid any spillage of urine, any remaining attachments divided and the entire specimen placed inside a laparoscopic bag.

For female cystectomy, placement of a blunt instrument in the vagina such as Rampley's sponge holders or a swab soaked in methylene blue, aid in identification of the vaginal apex after which a vaginal incision can be extended to the pelvic floor. The posterior vaginal plate can be closed on itself afterwards by intracorporeal suturing.

The left ureter is transposed to the right under the sigmoid mesocolon, bilateral lymphadenectomy is completed and the lymph node specimens are put in separate laparoscopic bags. The specimens can be delivered transvaginally in females, transrectally if a rectal pouch is being planned but most often by a 5–6 cm midline or muscle-splitting appendix incision.

### Reconstructive techniques

*The ileal conduit:* At this stage a number of reconstructive options exist. A urinary conduit may be created intra or extra-corporeally or a neobladder may be formed. The majority of centers appear to favour delivering the specimen through an oblique groin/appendix or subumbilical midline incision by extending a port-site incision to 5–6 cm. Through this an ileal conduit can be fashioned extra-corporeally. For ileal conduit formation, a 15–20 cm segment of terminal ileum is identified at least 25cm proximal to the ileocaecal valve. Linear stapling devices can be used to isolate the loop on its mesentery. The intestinal anastomosis can be constructed using a side-to-side technique with a linear stapler. The ileal loop is brought through the skin to a pre-marked stoma site. The ureters are then anastomosed to the ileal loop following spatulation using continuous absorbable sutures. Single J stents or infant feeding tubes splint the uretro-ileal anastomoses and the conduit is additional drained with a sump tube or Foley's catheter to reduce the risk of urinary leakage.

*Orthotopic neo-bladder:* Generally a neo-bladder can be constructed from 30–35 cm of ileum using a Z configuration or using a 65 cm segment for the Studer technique. Both ureters are directly re-implanted into the neo-bladder following spatulation. In the Studer neobladder, the proximal 10cm of ileum is left intact to form the afferent limb with the remainder being opened along the antimesenteric border. Continuous intra-corporeal suturing is used to create the neobladder itself after which it is anastomosed to the urethra with a running suture from the apex of the neobladder. Ureteric stents and a supra-pubic cystotomy catheter are introduced. This can be performed either extra or completely intracorporeally – the latter takes considerably longer and its benefits have to weighed against the downsides of a prolonged anaesthetic for the patient.

*Recto-sigmoid pouch (Mainz II):* This reconstruction involves incising the antimesenteric border of the colon and rectum approximately 10cm proximal and distal to the rectosigmoid junction. The posterior walls of the sigmoid and rectum are anastomosed side to side, forming the posterior wall of the pouch with a continuous absorbable suture. The mobilized ureters are brought through the completed pouch plate and secured with sutures using a submucosal tunnel to prevent reflux. Ureteric catheters are placed and externalised through the anus with a Foley's catheter to drain the pouch. The cystectomy specimen is brought out through the rectum prior to pouch formation. Pouchogram at 1–2 weeks confirms the pouch function and the stents and catheter are removed sequentially.

Perhaps the main hurdles to be overcome are the excessive operative time for the combined LRC and reconstruction with times up to 13.8 hours having been reported. Some centres are able to perform the initial LRC in 2–3 hours[17] with the reconstruction taking 2–3 hours afterwards. For this reason extra-corporeal reconstruction remains a popular option and the role of intracorporeal laparoscopic surgery remains debatable in this situation. (Table 2) details the advantages and disadvantages of LRC.

**Table 2:** Advantages and disadvantages of LRC

Outcome Criteria	Laparoscopic Radical Cystectomy	Open Radical Cystectomy
Blood Loss	Decreased (∼300 ml)	Significant (> 1 litre)
Post-operative pain	Decreased	Can be significant
Return to full activity Operative time	Weeks 5-13 hours Increased	Months 3-5 hours Reduced
Technical difficulty Cost	Highly advanced Expensive	Advanced Relatively cheaper
Long term outcome Cosmetic appearance	Unknown Smaller scars	Proven Vertical midline incision

With respect to oncological outcome, the data from the majority of the above series has yet to mature to beyond 2–3 years and many initial publications have focussed mainly on operative technique. Almost all reported cases had negative margins at the time of initial histological analysis with no reports of accidental perforation of the bladder intra-operatively. This probably reflects sound judgement in initial case selection for this novel technique. Extended lymphadenectomy can be achieved laparoscopically although it adds an hour to the operating time. Prostate sparing LRC is also being explored. This being said, the National Institute of Clinical Excellence UK (NICE) recently deemed the evidence for LRC to be inadequate as long term survival data is lacking.

At the time of writing, over 300 LRCs have been performed world-wide. The blood loss is about 300 ml with transfusion rates of 0–30%, decreasing with increasing experience.[1] The use of staplers reduces the blood loss and a randomised controlled trial of a linear stapler vs traditional ligation of pedicles in open radical cystectomy showed a lower blood loss in the stapler group.[26] The blood loss in LRC is even less due the additional tamponade effect of the pneumoperitoneum. Morphine requirements, post-operative ileus hospital stay and full recovery are significantly shorter than open surgery. Short term results of LRC are thus comparable to that of open cystectomy, although complication rates including mortality also appear to be similar. The Cleveland Clinic reported major and minor complication rates of 29% and 45% respectively.[3] Most patients with major complications need re-exploration while the minor complications can be managed conservatively. The mortality rate internationally is about 2%, rectal injury 2%, conversion 1.5% and intestinal obstruction 4–5%. In our experience we found that bowel obstruction from adhesions can be reduced by intraperitoneal instillation of icodextrin at the end of surgery.[1]

Simonato's recent article[17] looked at the three year follow up of the same 10 patients from his earlier paper of 2003.[27] In this updated report only two patients had metastatic disease; however two years later five were dead with the remainder free of clinical disease. They conclude that these results may be worse than those achieved with open surgery. In coming years further data should help resolve this worrying issue.

### Robotic radical cystectomy (RRC)

Following the success and consequent rapid expansion of robotic-assisted radical retropubic prostatectomy specialist centres began to examine the procedure of LRC to see if robotic assistance could confer a similar operative advantage.[28] The potential advantages of using the da Vinci Surgical System (Intuitive Surgical, Sunnyvale, Ca, USA) over LRC are improved intra-operative vision and increased dexterity. This may improve the accuracy of dissection and enhance the preservation of neurovascular bundles. Furthermore it may be more ergonomic and induce less fatigue in the console surgeon during a prolonged procedure.

The operative technique was recently described on 14 men and 3 women during collaboration by Menon's group from Detroit and the Mansoura Centre in Egypt.[29] The collective experience of having performed over 450 robotic radical prostatectomies and 3000 open cystectomies was clearly advantageous. Similar to LRC, it involves a six port trans-peritoneal approach but focuses on the initial development of the recto-vesical plane prior to transecting the recto-vesical pedicles. The procedure is in three stages; initially the pelvic lymphadenectomy and cystoprostatectomy, secondly extracorporeal formation of a neobladder and thirdly intra-corporeal urethro-neovesical anastomosis following re-docking of the robot. The operative times ranged from 260–310 min depending on whether an ileal conduit or orthotopic neobladder was formed. Blood loss was <150 mls and margins were clear in all cases. Beeken has described formation of a Hautmann orthotopic neobladder intra-corporeally using a da Vinci system with an operating time of 8.5 hours and a blood loss of 200 ml^30^, whilst Balaji's group from Nebraska have successfully performed a robotic-assisted totally intracorporeal laparoscopic ileal conduit urinary diversion.[31] The latter on 2 men and 1 woman had an operative time of 630–830 min with a hospital stay of 5–10 days. They highlighted the need for further operative refinement to reduce to prohibitive operative time. Menon's group have subsequently examined refined the robotic technique of LRC in women with preservation of the uterus and vagina. Urinary reconstruction was performed extracorporeally in the three-stage technique previously described.[32]

The Guy's technique of RRC and ileal conduit diversion has evolved from the Vattikuti RRC and Eastbourne LRC methods.[33,34] A team of surgeons experienced in robotic, laparoscopic and open radical cystectomy participate in each procedure. Posterior and lateral dissections are performed first with anterior dissection and control of the dorsal venous plexus left to the end. Ileal conduit formation is performed through a midline or appendix incision. Blood loss is on average 150 ml with hospital stay of 11 days. There have been no deaths so far. Patients over 60 are given prophylactic digoxin to prevent post-operative cardiac arrythmias. All oncological margins are negative and at 1 year follow-up no recurrences or metastases have occurred. A Robotic Steering Group has been established to scientifically evaluate the true benefit of robotics in urology and a randomized controlled trial of the three techniques of cystectomy is planned.[35,36]

### Laparoscopic partial cystectomy (LPC)

The indications for partial cystectomy are relatively few but include both malignant and benign disease. The main malignant indication is for single, primary, muscle-invasive, or high-grade bladder cancer that does not involve the bladder trigone, vesical neck, or posterior urethra and can be resected with adequate surgical margins. Partial cystectomy is also indicated with adenocarcinoma secondary to urachal duct remnants and a range of miscellaneous indications such as patient choice, and significant co-morbidity precluding radical cystectomy. Benign indications include resection of bladder diverticula, cavernous hemangiomas, ulcerative interstitial cystitis, colovesical fistula, vesicovaginal fistula, and localized endometriosis of the bladder.

With the global expansion of urological laparoscopy it will come as no surprise that this procedure can also be successfully performed laparoscopically. The initial case was performed for bladder endometriosis in 1994. Bladder resection was performed using the monopolar electrode and the bladder was then sutured laparoscopically.[37] The standard technique is to use absorbable sutures to close the defect and allow free catheter drainage of the bladder for at least one week. LPC has also been employed in the treatment of phaeochromocytoma,[38] schwannoma[39] and endocervicosis[40] of the urinary bladder. Gynaecologists specialising in laparoscopy have been confident in repairing both intentional (for endometriosis, vesico-vaginal fistula, embedded ovarian remnants) and unintentional cystotomies for several years.[41]

A group from Brazil have published the only series of partial cystectomy for transitional cell carcinoma of the bladder.[40] Using a harmonic scalpel for bladder resection and a two-layer closure, they report an operative time of 205 min with minimal blood loss with clear resection margins and no involved lymph nodes. One patient has required salvage chemotherapy in the 30 month follow-up. Despite this, laparoscopic partial cystectomy remains an uncommon procedure often performed unplanned by the gynaecologists during other operations or for rare bladder pathologies which can be carried out simply and effectively when indicated. There remains concern regarding tumour cell spillage and subsequent seeding in the uncontrolled environment during LPC for transitional cell carcinoma[43] and it is not generally recommended for this indication.

## CONCLUSIONS

At present, LRC remains a challenging procedure even for the skilled laparoscopist. With increasing laparoscopic experience and modifications of the technique it is likely that operative times and complications will reduce. Short term results have shown this to be a feasible technique that adheres to the basic principles of surgical oncology. However, the advantages of decreased blood loss, improved operative vision, reduced hospital stay and postoperative pain must be balanced against the increased technical difficulty, longer operative times, and unproved long-term oncological outcomes when compared with open radical cystectomy. Future studies need to look at the immunological changes after LRC and the effect of pneumoperitoneum on the biology of cancer cells.

Feasibility of the pure laparoscopic technique of LRC is now without doubt and the emphasis has moved to methods to reduce the prolonged time for reconstruction. Many of the published complications are related to the urinary diversion or neobladder rather than the laparoscopic cystectomy itself. Until surgeons are adequately skilled, many centers advise an extra-corporeal urinary diversion at present to reduce these risks and the operative time. With the advent of absorbable linear stapling devices and other technological advances in suturing devices the procedure may be simplified and the risk of staple-line stone formation reduced. Biotechnology advances could lead to the production of a new substrate with which to form the neobladder using tissue engineering techniques.

Longer-term oncological and functional follow-up data will be the main determinant of the eventual success of this exciting new procedure. Although LRC and RRC are here to stay, they will not be universally accepted unless cancer control, patient satisfaction and functional outcomes stand the test of time. Quality of life and cost issues are as yet not fully resolved.[1]

## References

[1] Rimington P, Dasgupta P (2004). Laparoscopic and robotic radical cystectomy. BJU Int.

[2] Nuttall MC, van der Meulen J, McIntosh G, Gillatt D, Emberton M (2005). Changes in patient characteristics and outcomes for radical cystectomy in England. BJU Int.

[3] Hrouda D, Adeyoju AB, Gill IS (2004). Laparoscopic radical cystectomy and urinary diversion: fad or future?. BJU Int.

[4] Parra RO, Andrus CH, Jones JP, Boullier JA (1992). Laparoscopic cystectomy: initial report on a new treatment for the retained bladder. J Urol.

[5] Zanetti G, Trinchieri A, Montanari E, Rovera F, Dell'Orto P, Taverna GL (1993). Laparoscopic cystectomy: an experimental model of urinary diversion Arch Ital Urol Androl. Arch Ital Urol Androl.

[6] Sanchez de Badajoz E, Gallego Perales JL, Reche Rosado A, Gutierrez de la Cruz JM, Jimenez Garrido A (1993). Radical cystectomy and laparoscopic ileal conduit. Arch Esp Urol.

[7] Puppo P, Perachino M, Ricciotti G, Bozzo W (1995). Laparoscopically assisted transvaginal radical cystectomy. Eur Urol.

[8] Denewer A, Kotb S, Hussein O, El-Maadawy M (1999). Laparoscopic assisted cystectomy and lymphadenectomy for bladder cancer: initial experience. World J Surg.

[9] Anderson KR, Fadden PT, Kerbl K, McDougall EM, Clayman RV (1995). Laparoscopic assisted continent urinary diversion in the pig. J Urol.

[10] Potter SR, Charambura TC, Adams JB, Kavoussi LR (2000). Laparoscopic ileal conduit: five-year follow-up. Urology.

[11] Gill IS, Fergany A, Klein EA, Kaouk JH, Sung GT, Meraney AM (2000). Laparoscopic radical cystoprostatectomy with ileal conduit performed completely intracorporeally: the initial 2 cases. Urology.

[12] Turk I, Deger S, Winkelmann B, Baumgart E, Loening SA (2001). Complete laparoscopic approach for radical cystectomy and continent urinary diversion (sigma rectum pouch). Tech Urol.

[13] Kaouk JH, Gill IS, Desai MM, Meraney AM, Fergany AF, Abdelsamea A (2001). Laparoscopic orthotopic ileal neobladder. J Endourol.

[14] Gill IS, Kaouk JH, Meraney AM, Desai MM, Ulchaker JC, Klein EA (2002). Laparoscopic radical cystectomy and continent orthotopic ileal neobladder performed completely intracorporeally: the initial experience. J Urol.

[15] Moinzadeh A, Gill IS, Desai M, Finelli A, Falcone T, Kaouk J (2005). Laparoscopic radical cystectomy in the female. J Urol.

[16] Cathelineau X, Arroyo C, Rozet F, Barret E, Vallancien G (2005). Laparoscopic assisted radical cystectomy: the montsouris experience after 84 cases. Eur Urol.

[17] Simonato A, Gregori A, Lissiani A, Bozzola A, Galli S, Gaboardi F (2005). Laparoscopic radical cystoprostatectomy: our experience in a consecutive series of 10 patients with a 3 years follow-up. Eur Urol.

[18] Arroyo C, Andrews H, Rozet F, Cathelineau X, Vallancien G (2005). Laparoscopic prostate-sparing radical cystectomy: the Montsouris technique and preliminary results. J Endourol.

[19] Yang S, Huang YH, Ou Yang CM, Huan SK, Chen M, Lin WR (2005). Clinical experience of laparoscopic-assisted radical cystectomy with continent ileal reservoir. Urol Int.

[20] Huang J, Xu KW, Yao YS, Guo ZH, Xie WL, Jiang C (2005). Laparoscopic radical cystectomy with orthotopic ileal neobladder: report of 33 cases. Chin Med J (Engl).

[21] DeGer S, Peters R, Roigas J, Wille AH, Tuerk IA, Loening SA (2004). Laparoscopic radical cystectomy with continent urinary diversion (rectosigmoid pouch) performed completely intracorporeally: an intermediate functional and oncologic analysis. Urology.

[22] Rimington PD, Rai SK, Adeniyi A (2003). Laparoscopic cystectomy and laparoscopic assisted ileal conduit: the initial experience. BJU Int.

[23] Gupta NP, Gill IS, Fergany A, Nabi G (2002). Laparoscopic radical cystectomy with intracorporeal ileal conduit diversion: five cases with a 2-year follow-up. BJU Int.

[24] Hemal AK, Singh I, Kumar R (2003). Laparoscopic radical cystectomy and ileal conduit reconstruction: preliminary experience. J Endourol.

[25] Hemal AK, Singh I (2004). Hand assisted laparoscopic radical cystectomy for cancer bladder. Int Urol Nephrol.

[26] Chang SS, Smith JA, Cookson MS (2003). Decreasing blood loss in patients treated with radical cystectomy: a prospective randomizes trial using a new stapling device. J Urol.

[27] Simonato A, Gregori A, Lissiani A, Bozzola A, Galli S, Gaboardi F (2003). Laparoscopic radical cystoprostatectomy: a technique illustrated step by step. Eur Urol.

[28] Tewari A, Peabody JO, Sarle R, Hemal AK, Shrivastava A, Menon M (2002). Technique of da Vinci robot-assisted anatomic radical prostatectomy. Urology.

[29] Menon M, Hemal AK, Tewari A, Shrivastava A, Shoma AM, El-Tabey NA (2003). Nerve-sparing robot-assisted radical cystoprostatectomy and urinary diversion. BJU Int.

[30] Beecken WD, Wolfram M, Engl T, Bentas W, Probst M, Blaheta R (2003). Robotic-assisted laparoscopic radical cystectomy and intra-abdominal formation of an orthotopic ileal neobladder. Eur Urol.

[31] Balaji KC, Yohannes P, McBride CL, Oleynikov D, Hemstreet GP (2004). Feasibility of robot-assisted totally intracorporeal laparoscopic ileal conduit urinary diversion: initial results of a single institutional pilot study. Urology.

[32] Menon M, Hemal AK, Tewari A, Shrivastava A, Shoma AM, Abol-Ein H (2004). Robot-assisted radical cystectomy and urinary diversion in female patients: technique with preservation of the uterus and vagina. J Am Coll Surg.

[33] Nicolson R, Dasgupta P, Khan S, Rimington P, Rose K (2005). Evolution of robotic cystectomy (Guy's experience). J Endourol.

[34] Rose K, Hemal AK, Cahill D, Rimington P, Khan MS, Dasgupta P (2005). Robotic radical cystoprostatectomy and ileal conduit diversion – first UK experience.

[35] Dasgupta P, Hemal A, Rose K (2005). Guy's and St. Thomas' Robotics Group. Robotic urology in the UK: establishing a programme and emerging role. BJU Int.

[36] Dasgupta P, Jones A, Gill IS (2005). Robotic urological surgery: a perspective. BJU Int.

[37] Ferzli G, Wenof M, Giannakakos A, Raboy A, Albert P (1993). Laparoscopic partial cystectomy for vesical endometrioma. J Laparoendosc Surg.

[38] Kozlowski PM, Mihm F, Winfield HN (2001). Laparoscopic management of bladder pheochromocytoma. Urology.

[39] Geol H, Kim DW, Kim TH, Seong YK, Cho WY, Kim SD (2005). Laparoscopic partial cystectomy for schwannoma of urinary bladder: case report. J Endourol.

[40] Nada W, Parker J, Wong F, Cooper M, Reid G (2000). Laparoscopic excision of endocervicosis of the urinary bladder. J Am Assoc Gynecol Laparosc.

[41] Nezhat CH, Siedman DS, Rottenberg H (1996). Laparoscopic Management of Intentional and Unintentional Cystotomies. J Am Assoc Gynecol Laparosc.

[42] Mariano MB, Tefilli MV (2004). Laparoscopic partial cystectomy in bladder cancer — initial experience. Int Braz J Urol.

[43] Andersen JR, Steven K (1995). Implantation metastasis after laparoscopic biopsy of bladder cancer. J Urol.

